# Identification of Differentially Expressed Proteins in the Serum of Colorectal Cancer Patients Using 2D-DIGE Proteomics Analysis

**DOI:** 10.1007/s12253-015-9991-y

**Published:** 2015-10-13

**Authors:** Lay Cheng Lim, Mee Lee Looi, Syed Zulkifli Syed Zakaria, Ismail Sagap, Isa Mohammed Rose, Siok-Fong Chin, Rahman Jamal

**Affiliations:** UKM Medical Molecular Biology Institute (UMBI), Universiti Kebangsaan Malaysia, Jalan Yaacob Latif, Cheras, 56000 Kuala Lumpur, Malaysia; School of Biosciences, Taylor’s University Lakeside Campus, Subang Jaya, Selangor, Malaysia; Department of Surgery, Faculty of Medicine, Universiti Kebangsaan Malaysia, Kuala Lumpur, Malaysia; Department of Pathology, Faculty of Medicine, Universiti Kebangsaan Malaysia, Kuala Lumpur, Malaysia

**Keywords:** Colorectal cancer, Proteomics, 2D-DIGE, LC-MS/MS, Apolipoprotein A1

## Abstract

Early detection of colorectal cancer (CRC) is vital for the improvement of disease prognosis. However to date there are no blood-based biomarkers sensitive and specific enough for early diagnosis. We analysed the differences in serum protein expression of early stage CRC (Dukes’ A and B) and late stage CRC (Dukes’ C and D) against normal controls using 2D Fluorescence Difference Gel Electrophoresis (2D-DIGE). Analysis of the 2D maps showed that 23 proteins were differentially expressed between groups (*p* ≤ 0.05) and these proteins were identified with LC-MS/MS. Eight proteins were up-regulated and 2 down-regulated in patients with early CRC, whereas 14 proteins were up-regulated and 4 down-regulated in those with late CRC compared to normal controls (*p* ≤ 0.05). Five proteins, namely apolipoprotein A1 (APOA1), apolipoprotein E (APOE), complement factor H (CFH), galectin-7 (GAL7) and synaptojanin-2 (SYNJ2) were validated using ELISA and only APOA1 and GAL-7 showed consistent findings. Further validation using immunohistochemistry showed negative immunoreactivity for GAL-7 in CRC tissues, suggesting that GAL-7 detected in the serum did not originate from the CRC tumour. APOA1 showed positive immunoreactivity but its expression did not correlate with Dukes’ staging (*p* = 0.314), tumour grading (*p* = 0.880) and lymph node involvement (*p* = 0.108). Differences in APOA1 isoforms and/or conformation between serum and tissue samples as well as tumour heterogeneity may explain for the discrepancies between DIGE and ELISA when compared to immunohistochemistry. Structural and functional studies of APOA1 in future would best describe the role of APOA1 in CRC.

## Introduction

Colorectal cancer (CRC) is the third most common cancer in men and the second in women worldwide [1]. In Peninsular Malaysia, it was ranked in 2006 as the most common cancer in men (16.2 % of the total cancer cases) and the second most common cancer in women (10.6 % of the total cancer cases) [2]. CRC is curable when detected at an early stage. It was reported that the 5-year survival rate is 90 % when CRC is detected at an early, localized stage; however, only 39 % of CRC are diagnosed at this stage due to the lack of specific and sensitive screening tests for early detection and monitoring of disease progression [3].

Current common screening tests for CRC include colonoscopy, flexible sigmoidoscopy, fecal occult blood test (FOBT) and double contrast barium enema [4]. Colonoscopy is the gold standard for CRC screening with 97 % sensitivity and 98 % specificity. However, it is not applicable to the general population due to its invasiveness, high cost, requires uncomfortable bowel preparation and highly trained medical personnel which leads to the reluctance of the general population at risk (>50 years) and those not at risk (<50 years) to go for this screening test. The remaining screening tests are hampered by their low sensitivities and specificities [5]. Hence, there is an urgent need to identify more sensitive and specific screening approaches which are less invasive and easy to perform for the early detection of CRC where therapy is most likely to be effective.

The use of serum biomarkers to distinguish between those with CRC and those without would be a useful tool in the CRC mass screening program since serum is one of the most accessible biological specimens which can be sampled. However, the serum also contains the widest dynamic range of cellular proteins in the body making it one of the most difficult biological fluids to be studied due to its complex nature [6]. It was reported that the serum proteome might reflect the health status of every organ and tissue in the body since most of the cells in the body are thought to leak and secrete proteins into the serum [6].

Although there have been many serum-based proteomic studies for CRC which have been published, none of the CRC biomarkers reported in the literature have gained FDA approval. Currently, the only protein biomarker which is clinically in use for the detection of CRC is carcinoembryonic antigen (CEA) [7]. However, it is not suitable as a screening biomarker due to its low sensitivity, specificity and positive predictive value [8]. It is widely used as a prognostic factor and recurrence indicator of CRC [9]. Due to the highly heterogeneous nature of CRC, it is likely that a panel of biomarkers instead of a single biomarker would be more effective in the early detection of CRC.

ColonSentry™, the world’s first commercially available blood-based test for CRC has exploited the multiple biomarkers approach in which a seven-gene blood-based mRNA biomarker panel is used to discriminate CRC [10, 11]. However the analysis of mRNA is not a direct reflection of the functional protein content in the cells. Poor correlation between mRNA and protein expression levels was reported in a previous finding [12] and it showed gene expression measurement at the mRNA level might not be sufficient. At cellular level, mRNA is subjected to post transcriptional modifications such as alternative splicing and this will result in functional alterations of the proteins. In addition, some mRNA might not be translated at all. Hence, there is still a strong basis for searching for proteomic-based biomarkers.

The aim of this study was to identify and validate potential protein biomarkers in the serum of patients with CRC. We used 2D Fluorescence Difference Gel Electrophoresis (2D-DIGE) coupled with tandem mass spectrometry (LC-MS/MS) and pathway analysis for identification of potential candidate protein biomarkers. These biomarkers were further validated using enzyme linked immunosorbent assay (ELISA) and immunohistochemistry.

## Materials and Methods

### Subjects Recruitment

This study was approved by the Medical Ethics Committee of the university and written informed consent was obtained from the patients. Complete clinical and pathological reports were obtained from the medical records.

Serum samples of newly diagnosed colorectal cancer patients from early stage (Dukes’ stage A and B) (*n* = 37) and advanced stage (Dukes’ stage C and D) (*n* = 27) were collected prior to surgery. Patients were recruited from 1st August 2008 until 31^st^ July 2010. Newly diagnosed patients were identified by their abnormal sigmoidoscopy or colonoscopy observation and confirmed by biopsy results. A total of 24 CRC adenocarcinoma serum samples were then selected randomly from the pool of patients and consisted of 8 each in the early stage, late stage and normal controls groups. Normal control subjects (*n* = 26) were recruited from the endoscopy clinic and these were patients who underwent colonoscopy and presented with benign colonic disease which included diverticular disease (diverticulosis and diverticulitis), colonic constipation and altered bowel habits. Patients who presented with polyps and ulcerative colitis were excluded from this study.

For the validation using immunohistochemistry, a total of 96 formalin-fixed paraffin embedded tissues (FFPE) of CRC patients from the years of 2005 to 2009 were retrieved from the Histopathology Unit, Department of Pathology. These cases included Dukes’ stage A (*n* = 2), Dukes’ stage B (*n* = 48), Dukes’ stage C (*n* = 37) and Dukes’ stage D (*n* = 9). The age of the patients ranged from 38 to 87 years (mean age, 65 years). Slides stained with haematoxylin and eosin were reviewed by a pathologist and a FFPE block with representative tumour was selected from each case for tissue sectioning.

### Serum Protein Enrichment

One mL of serum with estimated protein concentration of ≥50 mg/mL was used for protein enrichment by the ProteoMiner™ Protein Enrichment kit (BioRad, USA).

Sample clean-up was then performed on the enriched samples by using the ReadyPrep™ 2D clean-up kit (Biorad, USA) as per manufacturer’s protocols. The precipitated proteins were washed and resuspended in DIGE lysis buffer (9 M urea, 4 % CHAPS, 30 mM tris). The pH of the resuspended samples was adjusted between pH 8-9 by lysis buffer pH 11 prior to the CyDye labeling.

### Cy™Dye DIGE Fluor Minimal Labeling

After the enrichment, cleaning up and pH adjustment, the serum samples were labeled with the CyDyes (GE Healthcare, Uppsala, Sweden). A total of 5 nmol dry CyDye was reconstituted with 5 μL of anhydrous dimethylformamide (DMF) (Sigma Aldrich, USA). CyDye working solution (400 pmol/μL) was prepared by mixing the CyDye stock solution and DMF at 2:3 ratio. The samples were randomly labeled with the CyDye working solutions (Cy3 or Cy5) at a ratio of 400 pmol to 50 μg of proteins and were incubated for 30 min on ice. Cy2 was used to label a pooled sample (internal standard) which comprised of an equal amount of proteins from all samples. This was followed by quenching of the CyDye labeling activities by adding 1 μL of 10 mmol/L lysine to 50 μg of proteins for 10 min on ice. All procedures above were carried out in the dark.

### 2D Fluorescence Difference Gel Electrophoresis (2D-DIGE)

An equal volume of 2X sample buffer (8 M urea, 2 % IPG buffer, 130 mM dithiothreitol (DTT), 4 % CHAPS) were added to the pooled Cy2, Cy3 and Cy5 labeled samples (50 μg protein each) and were incubated for 10 min on ice in the dark. Rehydration buffer (8 M urea, 2 % CHAPS, 2 % IPG buffer, 20 mM DTT) was added to the samples to achieved a final volume of 340 μl. Samples were rehydrated overnight on 18 cm, pH 4-7 L Immobiline Drystrip gels and IEF was performed at 20 °C with a current limit of 50 μA/strip in the dark under the following conditions: step and hold for 2 h at 500 V, linear gradient for 3 h at 3500 V, 8000 V linear gradient to 15000 Vhrs, step and hold at 8000 V to 100,000 Vhrs for a total of 100 kVh. After first dimension separation based on proteins p*I*, the focused IPG strips were equilibrated with equilibration buffer I (6 M urea, 50 mM Tris–HCl pH 8.8, 30 % glycerol, 2 % SDS, 80 mM DTT) and equilibration buffer II (6 M urea, 50 mM Tris–HCl pH 8.8, 30 % glycerol, 2 % SDS, 169 mM IAA) for 15 min respectively before second dimension separation on a 1 mm-thick-10 % resolving gel. Gels were then visualized by using Ettan DIGE Imager (GE Healthcare) and then analyzed by ImageMaster 2D Platinum ver. 7.0, DeCyder 2D ver. 6.5 and DeCyder Extended Data Analysis (EDA) software (GE Healthcare).

### Protein identification by mass spectrometer

Gels were silver stained and the spots of interest were selected for trypsin digestion. Gel pieces were washed with 100 mM ammonium bicarbonate for 10 min. After washing, the gels were destained twice in solution containing 15 mM potassium ferricyanide and 50 mM sodium thiosulphate for 15 min each. Proteins were then reduced in 10 mM DTT in 100 mM ammonium bicarbonate for 30 min at 60 °C. The reduced proteins were then alkylated in 55 mM IAA in 100 mM ammonium bicarbonate for 20 min in the dark. Gels were washed with 50 % acetonitrile in 100 mM ammonium bicarbonate for 3 times, 20 min each. Gels were dried in speed vacuum for 15 min after the dehydration step with 100 % acetonitrile for 15 min. For protein digestion, 7 ng/μl trypsin solution was added and incubated at 37 °C overnight. Peptides were extracted with 50 and 100 % acetonitrile. Dried extract was reconstituted with 0.1 % formic acid solution and injected into the NanoACQUITY UPLC Q-TOF mass spectrometer (MS). MS was programmed to step between normal (5 eV) and elevated (25 to 40 eV) collision energies on the gas cell, using a scan time of 1.5 seconds per function over 50 to 1990 m/z. The ProteinLynx Global server (Waters, USA) was used as the search engine against human database Human and Enolase-1.0. Proteins identified were then subjected to pathway analysis by Ingenuity Pathway Analysis (IPA) software (California, USA).

Candidate biomarkers were selected from the list of differentially expressed proteins for validation with ELISA method. The selection criteria for these five biomarkers were based on: (1) protein functions which showed possible involvement in the CRC/cancer pathways from the literature review; (2) sequence coverage obtained by mass spectrometer with a value of ≥15 % (Table 1).

**Table 1:** Differentially expressed proteins identified by LC-MS/MS after DIGE analysis

No.	UniProt acc. number	Protein name	Sequence coverage (%)	Molecular weight (kDa)	pI	Early CRC vs. normal control	Late CRC vs. normal control	Biological process /molecular function(s)
1	Q15113	Procollagen C endopeptidase enhancer 1	19.4	47.9	7.3	Up	Down^*^	Proteolysis
2	Q53RT3	Human retroviral like aspartic protease 1	9.3	37	5.1	Up	Up^*^	Proteolysis
3	Q9P173	Human PRO2275	21.7	13.1	9.6	Up	Up^*^	Proteolysis
4	P68871	Hemoglobin subunit beta	73.5	16	6.9	Up	Up^*^	Transport proteins
5	Q363Q5	Truncated beta globin fragment	72.5	4.5	10	Up^*^	Down^*^	Transport proteins
6	P02768	Albumin	12.9	45.1	5.7	Up^*^	Up	Transport proteins
7	P02649	Apolipoprotein E	42.1	19.9	8.7	Up	Up^*^	Transport proteins
8	P02647	Apolipoprotein A1	31.1	30.8	5.4	Up^*^	Up^*^	Transport proteins
9	P47929	Galectin-7	52.2	15.1	7.6	Down^*^	Up	Apoptosis
10	C9JSK2	Human uncharacterized protein CFH	51.2	43.9	7.3	Up	Up^*^	Acute phase proteins
11	P08603	Complement factor H	32.1	31	7.7	Down	Down	Acute phase proteins
12	P0C0L4	Complement C4A	11.5	19.3	6.6	Up^*^	Up	Acute phase proteins
13	Q14624	Inter alpha trypsin inhibitor heavy chain H4	5.05	103.3	6.5	Up	Up^*^	Acute phase proteins
14	P01009	Alpha 1-anti trypsin	11.1	34.7	4.9	Up	Up^*^	Acute phase proteins
15	P02735	Human serum amyloid	20.2	25.4	6.1	Up	Up^*^	Acute phase proteins
16	P04070	Human vitamin K dependent protein C	21.7	52	5.8	Up	Up^*^	Coagulation factors
17	P00734	Human prothrombin	10	70	5.5	Up^*^	Up^*^	Coagulation factors
18	Q9GZN7	Protein rogdi homolog	12	32.2	8.3	Up^*^	Up^*^	Cellular proliferation & differentiation
19	Q70YC5	Human isoform 6 of protein ZNF 365	43.1	5.7	11	Down^*^	Down^*^	Cellular proliferation & differentiation
20	Q5SSB9	Ficolin collagen fibrinogen domain containing 3 hakata protein	39.4	11.4	4.6	Up^*^	Up	Signal transduction
21	O15056	Synatojanin-2	17.4	10	4.7	Up^*^	Up^*^	Signal transduction
22	P04262	Keratin type II cytoskeletal I				Down	Down^*^	Structural proteins
23		Human uncharacterized protein LOC134121	9.5	17	7.5	Up	Up^*^	Unknown

*Statistically significant differences (*p* < 0.05) when compared between different study groupss

### ELISA Validation

A total of 66 serum samples which comprised of 29 early stages of CRC, 19 late stages of CRC and 18 normal controls were selected for ELISA validation. Human apolipoprotein A1 (APOA1), apolipoprotein E (APOE), complement factor H (CFH), synaptojanin-2 (SYNJ2) and galectin-7 (GAL7) were validated using ELISA kits (Cusabio Biotech, USA) according to the manufacturer’s protocols. Briefly, 100 μl of standard, blank and samples were dispensed into the wells and incubated for 2 h at 37 °C. A total of 100 μl biotin-antibody working solution was added into each well and incubated for 1 h at 37 °C. One hundred μl of HRP-avidin working solution was added to each well and incubated for 1 h at 37 °C. This was followed by aspiration and washing for five times before 90 μl of TMB substrate added to each well and incubated for 30 min at 37 °C in the dark. Finally the reaction was stopped with the addition of 50 μl stop solution into each well and gently mixed for about 30 s. The microplate was then read at 450 nm within 30 min.

### Immunohistochemistry

CRC tissues (4 μm thickness) were mounted on L-polylysine slides. The sections were deparaffinized twice in Xylene and subsequently rehydrated with ethanol sequentially in descending concentrations of 100, 90, 70 and 50 % respectively. The sections were then washed before endogenous peroxidase quenching by 3 % H_2_O_2._ The sections were treated using a target retrieval antigen buffer, pH 9.0 (Dako, USA) in a decloaking chamber (pressure cooker) at 98 °C for 20 min. The sections were then cooled down at room temperature for 20 min. Thereafter, sections were incubated with the diluted primary antibody monoclonal mouse [12C8] anti-apolipoprotein A1 (4 μg/mL) for 30 min. The sections were then washed in TBS buffer 0.05 M, pH 7.6 for three times, 3 min each, and further incubated with secondary antibody from Dako Envision kit for 30 min. Sections were washed with TBS buffer 0.05 M, pH 7.6 again for 3 times, 3 min each. Sections were then developed for 8 min with high sensitivity diaminobenzidine (DAB) chromogenic substrate (Dako REAL™ Envision™ Detection System Peroxidase/DAB+, Rabbit/Mouse, Denmark) and counterstained with haematoxylin (Haematoxylin Solution Modified acc to Gill III, Merck, Germany). Finally, the sections were dehydrated sequentially with ethanol with descending concentrations of 50, 70, 90 and 100 % respectively. Sections were mounted with DPx (Merck, Germany) before viewed under light microscope.

### Statistical Analysis

Immunohistochemistry staining results were interpreted using a 2-tier scoring system by considering the area extent of staining, according to the scoring method by Kim et al. [13]. Every tumour was given a score according to the area extent of cytoplasmic stained cells: <10 % = 0; 10–50 % = 1; >50 % = 2. Results were analyzed by SPSS ver. 15.0 using Chi-square test. P-values less than 0.05 were considered as statistically significant.

## Results

A total of 2419 spots were detected by 2D-DIGE and 23 proteins (derived from 33 spots) were significantly differentially expressed between study groups (*p* ≤ 0.05) (Table 1). In the early stage CRC group, 8 proteins were up-regulated (truncated beta globin fragment, albumin, complement C4A, protein rogdi homolog, ficolin collagen fibrinogen domain containing 3 Hakata antigen, prothrombin, SYNJ2 and APOA1) and 2 proteins were down-regulated (GAL7 and isoform 6 of protein ZNF365).

In late stage CRC group, 14 proteins were up-regulated (hemoglobin subunit beta, human retroviral like aspartic protease 1, human uncharacterized protein CFH, human PRO2275, human uncharacterized protein LOC134121, APOE, human vitamin K dependent protein C, protein rogdi homolog, inter-alpha-trypsin inhibitor heavy chain H4, alpha 1-antitrypsin, human serum amyloid, prothrombin, SYJN2 and APOA1) and 4 proteins were down-regulated (PCOLCE1, truncated beta globin fragment, keratins and human isoform 6 of protein ZNF365) (Table 1).

The Ingenuity Pathways Analysis (IPA) revealed interaction between 21 proteins. These proteins are involved in cellular proteolysis, protein transport, apoptosis, acute phase reactions, coagulation factors, cellular proliferation and differentiation, structural protein and signal transduction pathways (Table 1).

**Fig. 1:**
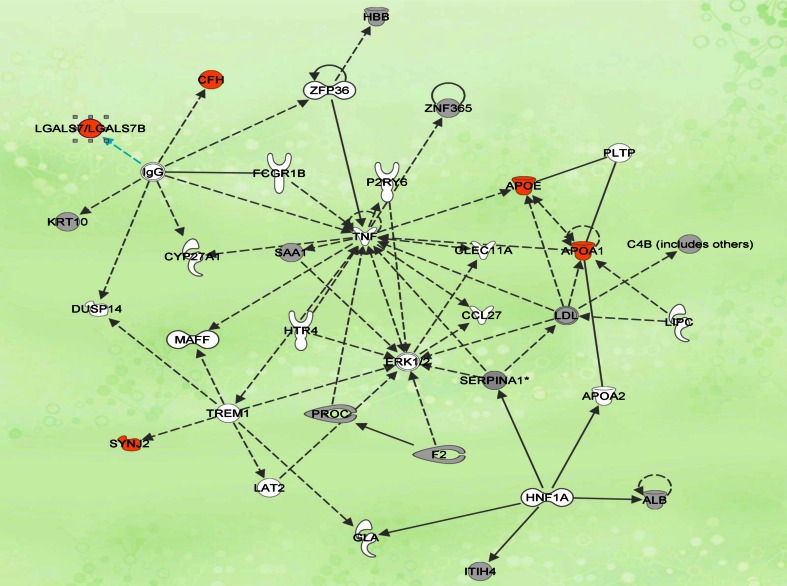
Biological interaction network revealed by Ingenuity Pathway Analysis. The network is revealed as circles (genes) and lines (biological relationship). *Solid lines* indicate direct interaction, and *dotted lines* indicate indirect interactions between the genes. Genes highlighted in *orange* are the potential candidates chosen for further validation

The top canonical pathways which are associated with CRC included liver X receptor/ retinoid X receptor (LXR/RXR) activation, acute phase response signaling, clathrin-mediated endocytosis signaling, blood coagulation and complement system pathway. Of these pathways, the LXR/RXR activation pathway is the most highly up-regulated pathway which also involves APOA1 and APOE regulation. The network is centered on TNF and ERK1/2 that regulate inflammatory responses, apoptosis and cell survival. Our pathway map showed the relationship between APOA1 and APOE with the TNF which acted as the central mediator in this network. CFH and GAL-7 were connected to TNF via IgG (Fig. 1). Hence, these four proteins were selected for further validation. In addition, SYJN2 was also chosen for further validation due to its reported role as novel Rac1 effector that regulates clathrin-mediated endocytosis pathway which plays a major role in malignant transformation and invasion [14]. It was reported that Rac1 activity is regulated by ERK-MAP kinase signaling [15].

**Fig 2:**
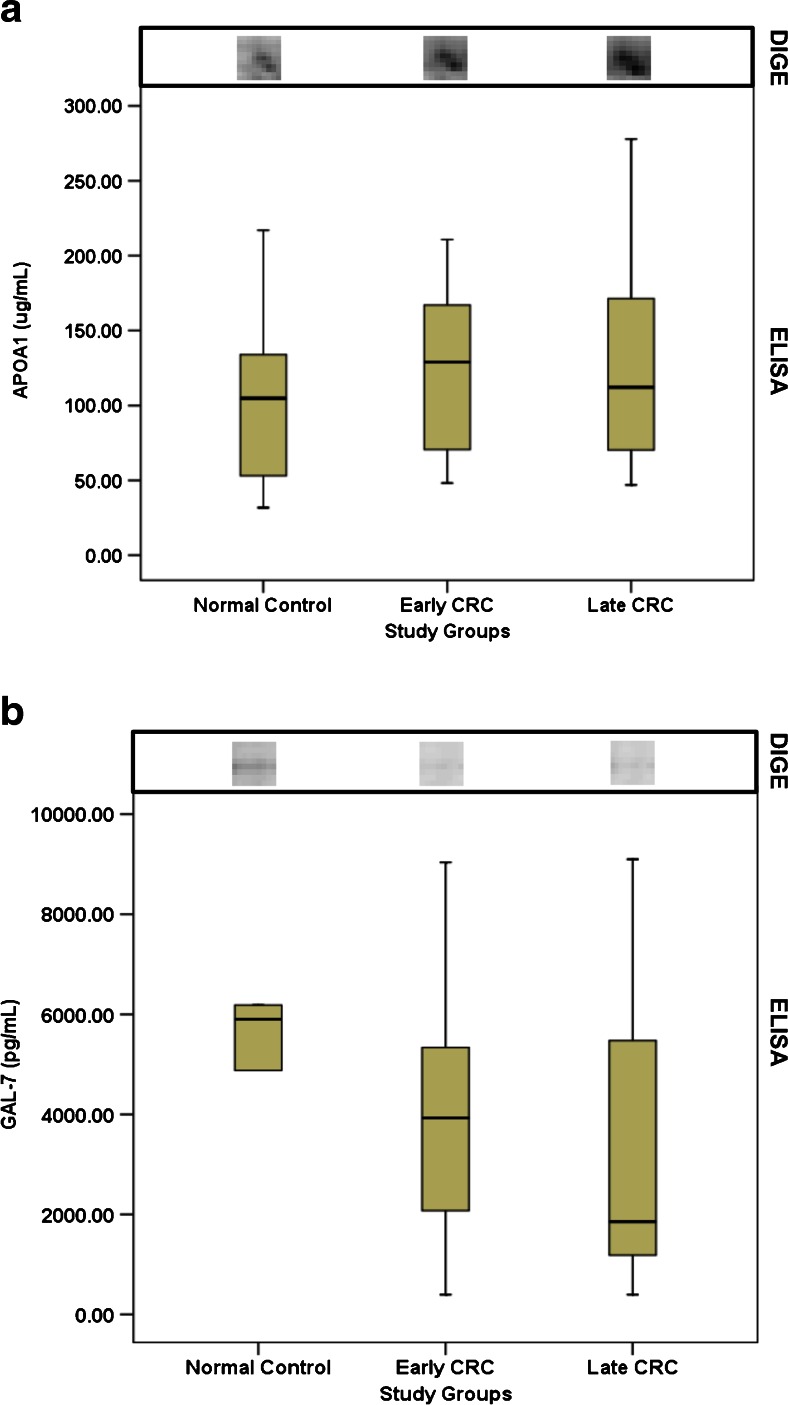
Expression of GAL-7 and APOA1 in serum of CRC patients. ELISA results shown by the boxplot. The spots for the respective proteins are depicted above the boxplot

Validation by ELISA showed APOA1 expression which is consistent with the DIGE findings both in normal controls versus early CRC and normal controls versus late CRC (Fig. 2a); whereas GAL-7 expression status was only comparable in normal controls vs. early CRC (Fig. 2b).

**Fig 3:**
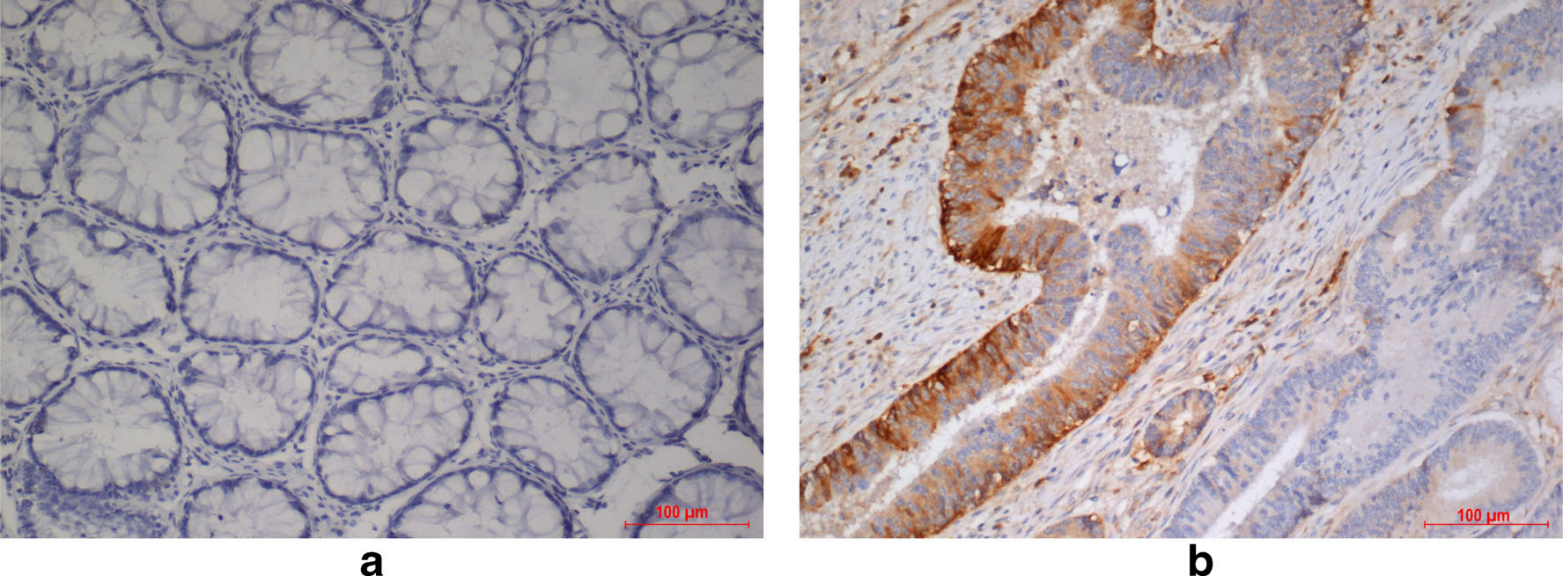
Immunohistochemistry staining of APOA1 in paraffin-embedded tissue of CRC. **a** Negative immunoreactivity in normal colonic cells (area stained score 0, ×200); **b** Cytoplasmic staining at glandular structure of CRC FFPE (area stained score 1, ×200)

APOA1 and GAL-7 was further validated at the tissue level using 96 FFPE blocks. Immunohistochemistry results showed 100 % negative immunoreactivity for GAL-7 in CRC tissues (data not shown). For APOA1, positive cytoplasmic staining was observed in 9 of 48 Dukes’ B, 12 of 37 Dukes’ C and 1 of 9 Dukes’ D (23 % of total CRC cases). Normal cells and Dukes’ A tumor cells did not show immunoreactivity of APOA1 (Fig. 3a). Specific cytoplasmic staining was observed in the tumour glands (Fig. 3b). APOA1 immunostaining showed no relationship between Dukes’ staging (*χ*2 = 4.86, *df* = 4, *p* = 0.30), tumour grading (*χ*2 = 1.19, *df* = 4, *p* = 0.88) and lymph node involvement (*χ*2 = 4.46, *df* = 2, *p* = 0.11).

## Discussion

We have identified 23 proteins which were differentially expressed between patients with colorectal cancer and those without. Our IPA analysis showed interaction between the two central mediators namely TNF and ERK1/2 (Fig. 1). The ERK pathway is one of the MAP kinase pathways that are activated by TNF and are mainly involved in regulation of cellular proliferation, differentiation, survival, apoptosis and stress responses and its dysregulation is a common occurrence in cancers [16]. The relationship between SYJN2 and Rac1 which were found to be regulated by ERK-MAP kinase signaling made us select this potential biomarker for ELISA validation [15]. SYJN2 is a novel Rac1 effector that is required for the formation of lamellipodia and invadopodia, as well as for tumour cell migration and invasion which makes it a novel potential target for therapeutic intervention in malignant tumours [17]. Lamellipodia and invadopodia are specialized membrane structures that are thought to be involved in extracellular matrix degradation.

In many cancers, IgG antibodies are produced that recognized cancer cells, form immune complexes and leads to the activation of the complement pathway [18]. CFH functions as a key regulator of the alternative pathway of the complement system [18]. The indirect interaction between IgG and GAL-7 from the network was based on a previous study which reported that IgG decreased the transcription of GAL-7 gene [19] via an unknown molecular mechanism [20]. We also selected GAL-7 for validation because of the reported novel function for GAL-7 in promoting tumorigenesis by upregulating MMP-9 gene expression [20].

The results of DIGE and ELISA were comparable in which both showed downregulation of GAL-7 in early CRC as compared to normal control and we investigated further GAL-7 expression at the tissue level. Galectins belongs to the family of beta-galactoside-binding proteins that are known to exhibit a pro-apoptotic function which is regulated through c-Jun N-terminal kinase (JNK) activation and mitochondrial cytochrome c release [21]. GAL-7 has also been reported to act as a pro-tumour protein which may be involved in the induction of matrix metalloproteinase-9 (MMP-9), which in turn plays an important role in cancer progression and metastasis [22, 23]. Our data showed GAL-7 to be down-regulated in early CRC suggesting its role in pro-apoptotic function. However, investigation of GAL-7 in CRC FFPE tissues revealed 100 % negative immunoreactivity in colorectal cancer tissues which implied that the GAL-7 detected in the serum might not be originating from the tumour tissues.

Cancer is also a disease of inflammation [24]. Inflammation has been found to induce the acute phase reaction, leading to multiple alterations in lipid and lipoprotein metabolism which are often induced by pro-inflammatory cytokines such as tumour necrosis factor (TNF) [25]. Cytokine-induced alteration of lipid metabolism during the acute phase response is achieved through the deregulation of type II nuclear hormone receptors which included liver X receptor (LXR) that will subsequently bind to DNA as obligate heterodimers with the retinoid X receptor (RXR) [26]. The LXR/RXR activation pathway plays an important role in regulating the gene transcription of APOE and ABCA1 which is responsible for APOA1 mediated cholesterol efflux [27, 28]. A previous study found that activation of LXR was able to inhibit the hedgehog signaling pathway which is an important regulator of tumour formation and carcinogenesis [29].

Our study showed that APOA1 was upregulated in early and late stage of CRC relative to normal controls both in DIGE and ELISA analysis. APOA1 is the major apoprotein constituent of high density lipoprotein (HDL) in plasma. The protein promotes cholesterol efflux from tissues to the liver for excretion and act as a cofactor for lecithin cholesterol acyl transferase (LCAT) which is responsible for the formation of most plasma cholesteryl esters. Besides its roles in the lipid transport, a recent study revealed that APOA1 has an important role in tumour invasion and metastasis in colonic adenocarcinoma [30]. There is one cohort study which showed the pre-diagnostic concentrations of APOA1 to be inversely associated with risk of colon cancer, but not rectal cancer [31]. One of the possible discrepancies of this study from our result could be due to the fact we include both colon and rectum cases in the analysis and that we had a much smaller sample size. Besides that, differences in the disease stage might have contributed to the discrepancies as well. The trend of APOA1 expression may be different in the pre-diagnostic stages of CRC as compared to post-diagnostic stages which generally included advanced tumour stages. Previous studies have reported similar observations as well where apolipoprotein CI (APOCI) in pre-diagnostic serum of breast cancer patients were found upregulated [32] but in another study using post-diagnostic serum samples, the upregulation was not observed in the [33]. In addition, yet another cohort study found that the biomarker panels discovered in diagnostic samples was not successfully validated in pre-diagnostic samples [34]. The molecular mechanisms of these differences need to be further elucidated.

Over-expression of APOA1 has also been observed in bladder cancer [35] and aggressive bladder transitional cell carcinoma [36]. Genetic variations of APOA1 may also act as a marker for the increased risk of breast cancer [37]. APOA1 is also part of the OVA1 test for ovarian cancer which utilizes APOA1, transthyretin, transferin, CA-125 and β2-microglobulin as the panel biomarkers [38].

In our study, further validation of the APOA1 at tissue level showed immunoreactivity in a proportion of samples but it did not show any relationship between Dukes’ staging, tumor grading and lymph node involvement. From our observation, the discrepancy in the results of DIGE and ELISA findings with immunostaining could be contributed by two factors: (1) differences in APOA1 isoforms and/or conformation between serum and tissues sample; (2) pronounced tumour heterogeneity.

APOA1 is the main protein component of HDL which is primarily synthesized in the liver and intestine. HDL exists in blood in two conformational forms: discoidal and spherical [39]. It was reported that the conformation of APOA1 in discoidal complexes is dependent on particle size and that these conformations are substantially different from that of APOA1 on spherical complexes [39]. We postulate that lipid-free APOA1 in the tissue might exist in different conformations and that the differences in APOA1 conformation may be critical to the antibody binding during immunochemistry assay. Hence, we postulate that this could explain the discrepancy between 2D-DIGE and ELISA versus immunostaining.

Tumour heterogeneity is a general phenomenon in cancer [40]. The tumour in colorectal cancer is often large and highly heterogeneous with marked stromal areas between glandular structures [41]. The conventional tumour sections resected for FFPE block only represent a very small fraction of the volume of most human tumours [42]. Hence it is possible that for some of the samples the FFPE block used might not adequately represent the tumour of origin.

In conclusion, our study has identified serum APOA1 as one of the potential biomarkers for CRC by showing consistent results with using ELISA. APOA1 needs to be further assessed structurally and functionally to provide more insight into its role in CRC.
